# Exploring the Use of Artificial Intelligence and Robotics in Prostate Cancer Management

**DOI:** 10.7759/cureus.46021

**Published:** 2023-09-26

**Authors:** Olumide Arigbede, Tope Amusa, Sarah G Buxbaum

**Affiliations:** 1 College of Pharmacy and Pharmaceutical Sciences, Institute of Public Health, Florida Agricultural and Mechanical University, Tallahassee, USA; 2 Oak Ridge Institute for Science and Education, Centers for Disease Control and Prevention, Atlanta, USA; 3 Department of Biostatistics, Georgia State University, Atlanta, USA

**Keywords:** patient outcomes, treatment decisions, algorithm, precision oncology, prostate cancer (pca), ai & robotics in healthcare

## Abstract

Integrating artificial intelligence (AI) and robotics in prostate cancer (PCa) offers a game-changing breakthrough with far-reaching implications for diagnosis, treatment, and research. AI-driven algorithms have tremendous promise for assisting early diagnosis by analyzing invisible trends within medical imaging devices such as MRI and ultrasounds. In addition, by evaluating big datasets containing patient data, genetic attributes, and treatment outcomes, these AI algorithms offer the possibility of allowing individualized treatment regimens. This ability to personalize actions to specific patients might improve therapy efficacy while reducing side effects. Robotics can increase accuracy in less invasive surgery, revolutionize therapies like prostatectomies, and improve recovery time for patients. Robotic-assisted procedures provide clinicians with remarkable skills and flexibility, allowing clinicians to negotiate complicated anatomical structures more precisely. However, the symbiotic combination of AI and robotics has several drawbacks. Concerns about data privacy, algorithm biases, and the need to continually assess AI’s diagnostic proficiency offer significant hurdles. To ensure patient privacy and data security, the ethical and regulatory aspects of integrating AI and robotics require proper attention. However, combining AI and robotics opens up a galaxy of possibilities. The joint use of AI and robotics can potentially speed up drug development procedures by filtering through massive databases, resulting in the identification of new medicinal compounds. Furthermore, combining AI and robotics might usher in an innovative era of personalized medicine, allowing healthcare providers to design therapies based on detailed patient profiles. The merging of AI and robotics in PCa care gives up unprecedented prospects. While limitations highlight the necessity for caution, the possibilities of better diagnostics, tailored therapies, and new research pathways highlight the transformational abilities of AI and robotics in determining the future of PCa management. This study explores the limitations and opportunities presented by using AI and robotics in the context of PCa.

## Editorial

Artificial Intelligence (AI) and robotics are not unfamiliar in the healthcare industry, particularly in cures for cancer. AI has helped to resolve a few multifaceted issues, including complex biological concerns like robotic surgery of prostatectomies-related tasks. AI anticipates playing significant roles in the future battle against cancers [1]. For over two decades, AI-based computer tools have assisted clinicians in interpreting mammograms [2]. Indeed, it is not an overstatement to say that AI and robotic sciences are transforming the healthcare sector, performing duties such as data collection, spotting tumor lesions, enhancing automated surgical procedures, finding cures for cancer, and giving enhanced therapeutic solutions [1,2]. AI is a mathematics and computer science field capable of developing tools to perform cognitive functions that currently need human intellect to attain a particular objective depending on the available data [1-4]. AI is reshaping the global healthcare landscape, while robotics empowers medical surgeons to perform highly intricate procedures with enhanced precision, reduced blood loss, less conspicuous scars for patients, shorter hospital stays, and increased patient satisfaction over time [1,4]. The current advancement of AI and robotics, such as the use of deep learning, mathematical algorithms, and the development of advanced medical systems, has resulted in the emergence of computer-based systems built to improve precision oncology and treatment decisions in cancer, particularly in prostate cancer (PCa) [3,4].

PCa develops in the prostate gland when cells multiply uncontrollably and spread rapidly, sustaining the abnormal cells as healthy cells die. Globally, PCa is the second most prevalent cancer in males, after lung cancer [5]. About 164,690 new cases in the United States were reported in 2018, accounting for 19% of new male cancer cases, with 29,430 PCa-related deaths (9% of male cancer fatalities) [3,6]. Incidence varies by race, with Black men having higher rates than White and Hispanic men [7]. Notably, the survival rates of PCa vary by race, with White men demonstrating longer survival rates than Black men. Several healthcare organizations across the globe are investigating, through scientific research, the root cause, prevention, identification, testing, and cure of PCa because of its burden on their health and economy. On average, the annual cost of PCa-related expenses exceeds $30,000 in the United States, placing a considerable strain on the healthcare system. It is important to note that as the population ages and economic activity increases, the prevalence of PCa is anticipated to increase, necessitating the need for improved implementation of AI and robotics [6].

The question is, can AI and robotics minimize economic costs and improve PCa patient outcomes? Yes, AI and robotics can reduce economic costs while improving PCa patient outcomes. In their study, Golla and Williams (2022) built a Markov model to assess the cost-effectiveness and economic benefits of robotic-assisted laparoscopic prostatectomy, open radical prostatectomy, and laparoscopic-assisted radical prostatectomy. The study showed that robotic-assisted laparoscopic prostatectomy was more cost-effective and affordable [8]. The level of adoption, the value of the tools, the cost of research and development, the cost of training personnel, the structure of healthcare facilities, and the frequency of technical improvements can all impact economic costs. AI and robotics can potentially increase efficiency, accuracy, and results in identifying, managing, and treating PCa [1,2,5]. Implementing AI and robotics in PCa might result in shorter hospitalization, fewer complications, enhanced care, and more efficient use of assets, all of which could lead to cost savings and better patient outcomes [5,6,9].

The use of AI and robotics cannot be overemphasized in oncology, and they can potentially transform the management and cure of PCa altogether. In addition, AI and robotics usage in PCa includes the following: (1) They assist in the early identification and precise diagnosis of PCa. Developing mathematical algorithms can help assess medical imaging systems, such as ultrasound images, and spot insignificant trends and discrepancies that would be impossible to detect with the human eyes [2]. (2) AI may be used to attain precision oncology. AI-assisted individualized treatment plans for each patient according to their distinct genetic profile, past health history, and other indicators can result in personalized and reliable therapeutic approaches, thereby reducing unnecessary treatments and adverse effects [1]. In addition, AI built from proven machine learning algorithms, predictive models, and imaging analysis can help surgeons construct individualized surgery strategies, clearly visualize prostate tumor surroundings, and foresee emerging complications for each patient [1,2]. (3) Robotic innovations have the potential to modify norms of invasive PCa surgery by improving treatment results, patient satisfaction, surgery time, flexibility, and accuracy for specialists in surgery, facilitating fewer incisions, fewer bleeds, and faster recovery periods for patients [1,3,5]. (4) AI can evaluate big databases of patient data, treatment results, and other medical data to anticipate how alternative therapeutic options would work for specific patients. Thus, AI can help determine the best treatment technique to increase the likelihood of survival [3,5]. (5) AI systems can help PCa patients schedule therapy sessions such as radiotherapy. AI may help identify appropriate radiation dosage allocation by assessing the patient’s physiology and tumor characteristics, avoiding harm to healthy tissues while successfully targeting the tumor [3,4]. (6) AI-powered robots can help follow up PCa patients. AI-powered robots can continually track patients following treatment, recognizing any symptoms of relapse or issues as soon as they appear. AI-powered robots can allow for quicker intervention and better patient outcomes. In communities with limited resources, AI-guided clinical treatment can significantly reduce health disparities [10]. (7) AI can enhance cancer screening, help in tumor genome profiling, expedite drug discovery, and improve cancer surveillance. AI and robotics can also help with research and drug development. AI can aid with developing novel medications or recycling existing drugs for the treatment of PCa by evaluating vast volumes of biological and chemical data [2-4,9,10].

Employing AI and robotics in PCa management, education, and research might be beneficial. AI and robotics have the potential to successfully complement each other in PCa research, encompassing areas such as diagnosis, therapy, intervention, and discovering the underlying causes of the disease. The collaborative use of AI and robotics in PCa care and research can help collect precise imaging data, such as microscopy imagery, and analyze massive volumes of complicated medical data, including genomic data. These data can then be analyzed to identify modest patterns, mutations, and associations that human researchers may miss and uncover physiological or molecular alterations suggestive of PCa [1-9]. In executing intricate duties usually delegated to trained professionals, computer-based decision-making systems built using AI tools can revolutionize medical oncology by increasing diagnostic precision, boosting operational efficiency, enhancing clinical procedures, lowering administrative expenses, and improving treatment options. With rising applications in diagnostic imaging, surgical treatments, skill development and evaluation, computerized pathology, and genomics research, these traits may be very beneficial in managing PCa. In addition, the combination of AI and robotics can aid in analyzing biopsy samples and automating the tissue collection procedure. These are designed to help in the identification of malignant cells, the determination of PCa subtype or stage, the reduction of disease risk, and the provision of consistent samples for analysis. As heavy users of imaging and pathology, clinicians, including urologists, oncologists, and others, it is recommended to understand this developing field of study and accept that collaboration with data scientists, technologists, bioinformaticians, computer researchers, and engineers will be necessary to create highly accurate AI-based decision-support applications and robotics [2,4].

The utilization of AI and robotics in PCa care raises various ethical concerns and limitations. Ethical concerns surrounding the use of AI and robotics in PCa include the potential for algorithmic biases and the development of PCa disparities. In the context of PCa management, if historical data used to train algorithms contains biases related to race, income level, or geographical region, the resulting AI systems may unintentionally perpetuate the biases existing in the data they are trained on. Algorithm biases might result in differences in treatment recommendations for different patient groups, thus exacerbating disparities in PCa. Furthermore, socioeconomic differences in access to cutting-edge therapies can exacerbate PCa disparities. Patients with limited resources or those living in impoverished regions may have less access to facilities that offer robotic surgery or AI-powered diagnostic tools, resulting in discrepancies in the quality of treatment they receive [11]. The inability of AI and robotics to imitate human reactions, moral or ethical reasoning, and the possibility of decision-making errors, which can have substantial social and safety consequences, are among the limitations of AI and robotics [11,12]. Because AI algorithms require high-quality data to make accurate predictions and analyses to build reliable robots, it is recommended that the data used to train AI models be comprehensive, diversified, and representative of various patient groups [12].

Given the limitations and ethical concerns described above, it is imperative that AI models, like any other models, undergo a comprehensive evaluation process. Importantly, prioritizing patient confidentiality and data safety by following applicable rules such as the Health Insurance Portability and Accountability Act can help build trust between patients and clinicians [1]. Furthermore, before being widely used, AI and robotics systems should be subjected to thorough clinical trials and testing to verify their reliability, effectiveness, safety, and influence on patient outcomes [4,11]. Encouraging partnerships among healthcare institutions, AI developers, and regulatory bodies is crucial to collectively addressing ethical concerns and limitations. This partnership can help ensure the ethical integrity and efficacy of AI and robotics technologies in PCa care [4,11]. Lastly, AI algorithms should be evident and understandable, allowing physicians to comprehend how the AI arrived at its results [3,9,11]. Understanding AI algorithms may contribute to developing trust among healthcare providers and facilitate the integration of AI guidance into healthcare decision-making. As machine learning and robotics technology evolve, AI and robotics may be enhanced by developing highly intelligent and reliable robots that adapt to changing surgical environments, thereby propelling human efforts toward conquering PCa.
